# Identification of novel key genes associated with the metastasis of prostate cancer based on bioinformatics prediction and validation

**DOI:** 10.1186/s12935-021-02258-3

**Published:** 2021-10-25

**Authors:** Feifeng Song, Yiwen Zhang, Zongfu Pan, Xiaoping Hu, Yaodong Yi, Xiaochun Zheng, Haibin Wei, Ping Huang

**Affiliations:** 1 Clinical Pharmacy Center, Department of Pharmacy, Zhejiang Provincial People’s Hospital, Affiliated People’s Hospital, Hangzhou Medical College, Hangzhou, 310014 Zhejiang China; 2 Key Laboratory of Endocrine Gland Diseases of Zhejiang Province, Hangzhou, 310014 Zhejiang China; 3 Laboratory of Drug Metabolism and Pharmaceutical Analysis, College of Pharmaceutical Sciences, Zhejiang University, Hangzhou, 310058 China; 4 Department of Urology, Zhejiang Provincial People’s Hospital, People’s Hospital of Hangzhou Medical College, Hangzhou, 310014 Zhejiang China

**Keywords:** Prostate cancer, Metastasis, Differentially expressed genes (DEGs), Key genes, TCGA, EMT, Bioinformatics analysis

## Abstract

**Background:**

Metastatic prostate cancer (PCa) is a lethal tumor. However, the molecular mechanisms underlying PCa progression have not been fully elucidated.

**Methods:**

Transcriptome expression profiling and clinical information on primary and metastatic PCa samples were obtained from TCGA. R software was used to screen the DEGs, and LASSO logistical regression method was utilized to identify the pivotal PCa metastasis-related DEGs. The transcriptional expression levels of the key genes were analyzed using the UALCAN database, and the corresponding protein expression were validated by Immunohistochemistry (IHC). Survival analysis of the key genes was performed using the GEPIA database. Wound healing assay and Transwell assay were conducted to determine whether knockdown of the key genes influence the migration and invasion abilities of PCa cells (22Rv1 and PC3). GSEA was performed to predict key genes-mediated signaling pathways for the development of PCa. Western blotting was used to evaluate the expression changes of E-cadherin, Twist1, and Vimentin in PCa cells with the key genes silencing. An in vivo mouse metastatic model for PCa was also generated to verify the important role of ISG15 and CST2 in PCa metastasis.

**Results:**

A comparison between primary and metastatic PCa tissues was conducted, and 19 DEGs were screened. Among these, three key genes were identified that might be closely associated with PCa progression according to the LASSO logistical analysis, namely ISG15, DNAH8, and CST2. Further functional experiments revealed that knockdown of ISG15 and CST2 suppressed wound healing, migration, and invasion of PCa cells. To explore the molecular mechanism of ISG15 and CST2 in the development of PCa, GSEA was performed, and it was found that both genes play crucial roles in cell adhesion molecules, extracellular matrix-receptor interaction, and focal adhesion. Western blotting results exhibited that inhibiting ISG15 and CST2 led to increase the expression of E-cadherin and decrease the expression of Twist1 and Vimentin. Additionally, the metastatic in vivo study demonstrated that both PC3 and 22Rv1 cells expressing with luciferase-shISG15 and luciferase-shCST2 had significantly lower detectable bioluminescence than that in the control PCa cells.

**Conclusion:**

ISG15 and CST2 may participate in PCa metastasis by regulating the epithelial-mesenchymal transition (EMT) signaling pathway. These findings may help to better understand the pathogenetic mechanisms governing PCa and provide promising therapeutic targets for metastatic PCa therapy.

**Supplementary Information:**

The online version contains supplementary material available at 10.1186/s12935-021-02258-3.

## Background

Prostate cancer (PCa) is the second most frequently diagnosed cancer and the fifth leading cause of cancer-related deaths among men worldwide [1]. According to the latest global cancer statistics, new cases of PCa are estimated to reach 1.4 million, and the number of new deaths is approximately 375,304 in 2020 [1]. Although significant improvements have been made in the early diagnosis of PCa, about 16% of men with PCa in the Netherlands have progressed to an advanced stage at first diagnosis [2]. Moreover, the incidence rate of metastatic PCa is predicted to increase by nearly 42% in the United States by 2025 [3]. Patients with metastatic PCa usually have a poor prognosis, with a 5-year survival rate of as low as 29% [3]. Currently, androgen deprivation therapy (ADT) is the first line treatment for newly diagnosed metastatic PCa, such as abiraterone and enzalutamide. However, the response of patients with metastatic PCa to ADT is only maintained in a short term, and almost all patients develop resistance to ADT therapy within several years [4, 5]. Chemotherapy or radiotherapy, such as mitoxantrone and docetaxel, are commonly adopted for ADT-nonresponsive or metastatic PCa patients, but their efficacy in extending recurrence and decreasing toxicity is limited [6, 7]. Therefore, there is an urgent need to identify novel molecules associated with PCa metastasis and understand the underlying molecular mechanism, which will be developed as potential therapeutic targets for metastatic PCa therapy.

Bone is the most common metastatic site in advanced PCa, and bone metastasis is often accompanied by a series of complications such as fracture and intractable pain, which dramatically reduces patients’ quality of life [8, 9]. Tumor metastasis is a complex process that involves multiple steps, including tumor cell dissociation from the primary site, local invasion, intravasation, transport and survival in circulation, extravasation to new sites, and colonization [10]. Increasing studies have demonstrated that genetic alterations, epigenetic modifications, and tumor microenvironment play crucial roles in the metastatic process of PCa. Inherited mutations in *BRCA2*, *ATM*, *CHEK2*, *BRCA1*, *RAD51D*, and *PALB2* have been identified as important prognostic factors for PCa development [11]. Based on high-density microarrays, Stankiewicz et al. have found that *FBXL4* is a suppressor gene of PCa, which is lost during PCa progression [12]. Depending on the gene expression profiling of metastatic PCa samples, Varambally et al. have revealed that enhancer of zeste homolog 2 (a histone lysine methyltransferase enzyme) was upregulated in advanced PCa, and its high expression was related to the progression of PCa [13]. Additionally, the tumor microenvironment, especially tumor-associated inflammation, is also significantly correlated with disease development. A recent study has pointed out that CXCL1 could interact with cytokines from neutrophils and promote epithelial-mesenchymal transition (EMT), thereby accelerating PCa metastasis [14]. However, despite great advancements in identifying the pivotal genes for advanced PCa, its specific metastasis-associated genes and underlying mechanisms are not completely understood.

In this study, we analyzed the transcriptome expression profiles from The Cancer Genome Atlas (TCGA) database and screened the DEGs between primary tumors and advanced PCa. Three key genes (ISG15, DNAH8, and CST2) were identified that were particularly correlated with metastasis according to the LASSO logistic regression. Furthermore, the expression alterations of the three key genes during disease progression were obtained from UALCAN and validated by Immunohistochemistry (IHC). Moreover, prognostic analysis was performed using the GEPIA database. To better understand the role of these key genes in metastatic PCa, we conducted wound healing assay and Transwell assays to investigate the migration and invasion abilities of PCa cells with or without key genes knockdown. Additionally, to further clarify the molecular mechanism of these key genes mediating PCa metastasis, gene set enrichment analysis (GSEA) was applied to perform the pathway enrichment analysis, and western blotting was utilized to validate the expression alterations of genes in enriched pathway. Furthermore, an in vivo metastatic mouse model was generated to verify the important role of key genes in PCa metastasis. By comparing primary and advanced PCa, our study aimed to identify novel key genes associated with metastasis and understand the underlying mechanism, which may provide potential targets for advanced PCa therapy.

## Materials and methods

### Data collection and processing

The transcriptome expression profiles and corresponding clinical information of prostate tissue samples were downloaded from TCGA database (https://portal.gdc.cancer.gov/). To screen the DEGs responsible for PCa metastasis, the prostate samples were divided into primary prostate adenocarcinoma samples (n = 310) and metastatic prostate adenocarcinoma samples (n = 73). Before the analysis of TCGA data, transcriptome data (FPKM values) were transformed into TPM values [15]. After normalization, a comparison between primary and metastatic samples was conducted using the limma package from R software. DEGs were then identified based on the thresholds as |log_2_(fold change)|> 1.0 and adjusted P value (adj.P.Val) < 0.05.

### Transcriptional expression analysis

Based on the LASSO logistic regression, the expression of DEGs in primary and metastatic PCa was conducted using the ggbeeswarm package from R software. After identifying the potential key genes, the transcriptional levels of these genes were analyzed using the online UALCAN (http://ualcan.path.uab.edu/) database. UALCAN is an online resource for gene expression and survival analyses in various tumors, providing easy access to public transcriptional expression data, including TCGA [16]. In the present study, UALCAN database was applied to analyze the transcripts of these key genes in normal, primary, and metastatic prostate adenocarcinoma tissues. P value was shown in the webpage, and P < 0.05 was considered statistically significant.

### Clinical prostate sample collection

Paraffin-embedded prostate tissues of primary and metastatic PCa were obtained from patients who had undergone surgery between 2018 and 2020 at Zhejiang Provincial People’s Hospital (Hangzhou, China). The tissues were fixed in paraffin and stored in the pathology department of Zhejiang Provincial People’s Hospital. A total of three primary prostate tissue samples and three metastatic prostate tissue samples were obtained, and the corresponding clinical information were listed in Additional file 1: Table S1. This study was approved by the Institutional Review Board of Zhejiang Provincial People’s Hospital (IRB-2021019).

### Immunohistochemical staining

Paraffin-embedded prostate sections were deparaffinized with xylene and rehydrated with ethanol. Then the sections were treated with 1 mM EDTA to retrieve the antigen and preincubated with 5% goat serum in TBS to decrease non-specific binding. Next, the sections were incubated with the following primary antibodies: anti-ISG15 (Cat. 15981-1-AP, 1:200), anti-CST2 (Cat. 19935-1-AP, 1:20) (Proteintech, Wuhan, China), anti-DNAH8 (Cat. HPA028447, 1:250, Sigma, Oakville, Canada). After incubation with horseradish peroxidase secondary antibodies, the prostate sections were measured using DAB (Beyotime, Hangzhou, China) and counterstained with hematoxylin.

The IHC score included immune-positive rate and the staining intensities of each sample. In the present study, the protein expressing score of all the slices were analyzed by the pathologist. The immune-positive rate was graded from 0 to 4, where less than 5% of cells stained was scored as 0; 6–25% was scored as 1; 26–50% was scored as 2; 51–75% was scored as 3; and 76–100% was scored as 4. The staining intensities were graded from 0 to 3, where 0 was defined as negative; 1 as weak; 2 as moderate; and 3 as strong. The protein score of ISG15, CST2 and DNAH8 was calculated as the product of intensity and positive rate, which ranged from 0 to 12.

### Survival analysis

Survival analysis of the key genes in patients with prostate adenocarcinoma was conducted using the GEPIA database (http://gepia.cancer-pku.cn/) [17]. GEPIA is an online tool used to perform the differential expression analysis, correlation analysis, and survival analysis based on the TCGA and GTEx projects. In this study, survival curves including overall survival (OS) and disease-free survival (DFS) were generated based on high and low expression of key genes. The cutoff value was set as quartile, and P < 0.05 was considered statistically significant.

### Gene set enrichment analysis

To investigate the signaling pathways of the key genes participating in the metastasis of prostate adenocarcinoma, GSEA was performed using R software (version 4.0.3) and clusterProfiler package (version 3.18.0). The clusterProfiler package was used for biological-term classification and enrichment analysis of gene clusters [18]. For GSEA, the KEGG enrichment maps were generated based on high and low expression of key genes.

### Cell culture

Human PCa cell lines 22Rv1 and PC3 were purchased from the Type Culture Collection of the Chinese Academy of Sciences (Shanghai, China). 22Rv1 and PC3 cells were cultured in Roswell Park Memorial Institute (RPMI) 1640 medium (Gibco, USA) containing 10% fetal bovine serum (Gibco, USA) and 1% penicillin/streptomycin (Sangon Biotech, Shanghai, China) and maintained at 37 °C in a 5% CO_2_ incubator.

### Construction of shISG15 and shCST2 PCa cell line

Lentivirus encoding the shRNAs were designed and synthesized by HANBIO (Shanghai, China) and the sequences of shRNAs were listed in Additional file 1: Table S2. According to the manufacturer’s instructions, 22Rv1 and PC3 cells were infected with lentivirus expressing Luc-shCtrl, Luc-shISG15 or Luc-shCST2 for 48 h and selected with puromycin (Cat. ant-pr-1, InvivoGen, USA) at a concentration of 4 µg/mL.

### RNA extraction and qRT-PCR

After generation of the indicated stable cell line, PCa cells were collected and total RNA was extracted using the AxyPrep Multisource RNA Miniprep Kit (Cat. AP-MN-MS-RNA-250, Union City, USA). cDNA was generated by using HiScript II Q RT SuperMix (Vazyme, Cat. R222-01, Jiangsu, China). Next, TB Green *Premix Ex* Taq II (Takara, Cat. RR820Q, Kusatsu, Japan) was used to prepare the quantitative polymerase chain reaction amplification reaction. The mRNA expression of target genes was determined by qRT-PCR using the LightCycler480 II system (Roche, IN, USA) and quantified by the 2^−ΔΔCt^ method. Primers for qRT-PCR were purchased from Sangon Biotech (Shanghai, China) and are listed in Additional file 1: Table S3.

### Western blot

Cells were collected and lysed in RIPA buffer containing 1 mM PMSF (Beyotime, Cat. ST506, Shanghai, China). Protein samples were denatured at 100 °C for 10 min and then separated by 10% SDS-PAGE. After being transferred to a PVDF membrane (Millipore, Cat. IPVH00010, Bedford, USA), 5% skim milk in TBST was used to block non-specific binding for 2 h at room temperature. Then the membrane was incubated with the following primary antibodies: anti-CST2 (Cat. 19935-1-AP, 1:1000), anti-ISG15 (Cat. 15981-1-AP, 1:250), anti-E-cadherin (Cat. 20874-1-AP, 1:1000), anti-Twist1 (Cat. 13099-1-AP, 1:500), anti-Vimentin (Cat. 10366-1-AP, 1:10,000), and anti-GAPDH (Cat. 60004-1-Ig, 1:10000) (Proteintech, Wuhan, China) at 4 °C overnight. Next day, membranes were washed with TBST for three times and incubated with the secondary antibodies conjugated with horseradish peroxidase, goat anti-rabbit (Cat.70-GAR0072, 1:5000) or goat anti-mouse (Cat.70-GAM0072, 1:5000) (Lianke Multi Sciences, China), at room temperature for 1 h. ECL (Cat.20-500-120, Beit HaEmek, Israel) was used to detect the binding antibodies by ChemiDoc™ XRS^+^ System (Bio-Rad, USA).

### Wound healing assay

After the stable PCa cells grown to be confluent, a micropipette tip was applied to make a cross wound. Then cells were washed with PBS for three times and cultured in serum-free RPMI-1640 medium and Mitomycin C (5 µg/mL, Cat. M5353, Sigma, Oakville, Canada). Photographs were taken by microscopy at once and after wounding for 24 h.

### Transwell assay

Cell migration and invasion assays were conducted using 24-well transwell chambers (8 µm pore size, Corning, Cat. 8432, USA). For a cell invasion assay, the inserts were precoated with 250 μg/mL Matrigel (BD Bioscience, Cat. 356234, USA) on the upper surface and incubated at 37 °C for 2 h. For migration and invasion assays, about 1 × 10^5^ cells were suspended in 0.2 mL serum-free RPMI-1640 medium containing 5 µg/mL Mitomycin C and added to the inserts. The lower compartment of the chamber was filled with 0.6 mL of RPMI-1640 containing 10% fetal bovine serum. After incubation for 24 h, the cells on the upper membrane were carefully removed using a cotton bud. Cells that invaded the chamber membranes were fixed with 4% paraformaldehyde and stained with 0.1% crystal violet. Five microscopic views of 100 × magnification of each insert was selected randomly under a phase-contrast microscope (Olympus, Japan) and counted using ImageJ 1.52a (Wayne Rasband, USA).

### In vivo animal study

4–6 weeks old BALB/c nude male mice (20 ± 2 g) were purchased from Beijing Vital River Laboratory Animal Technology Co., Ltd. All the animal experiments were approved by the Institutional Animal Care and Use Committee of Zhejiang Provincial People’s Hospital (A202100033). Mice were kept at least one week before cell implantation. For metastatic in vivo model, BALB/c nude mice were randomized into six groups: 22Rv1-Luc-shCtrl, 22Rv1-Luc-shISG15, 22Rv1-Luc-shCST2, PC3-Luc-shCtrl, PC3-Luc-shISG15, and PC3-Luc-shCST2. Intravenously (I.V.) injection was performed in this study as previously reported [19]. Briefly, each group of PCa cells (5 × 10^5^) were resuspended in 100 µL PBS and slowly injected into the tail vain of the mice. After injection, the IVIS imaging system (IVIS Lumina Series III, Perkin Elmer) was used to monitor and visualize the distribution of tumor cells. D-luciferin (Cat. 7903, DAKEWE, Shenzhen, China) at 150 mg/kg was injected in mice 10 min prior to IVIS spectrum imaging. Tumor burden was measured based on total photons per second with background subtraction per region of interest (ROI).

### Statistical analyses

All data are expressed as the mean ± SD. Statistical comparisons between the two groups were determined by Student’s unpaired two-tailed t test. P < 0.05 was considered statistically significant.

## Results

### Identification of DEGs associated with PCa metastasis

**Fig. 1:**
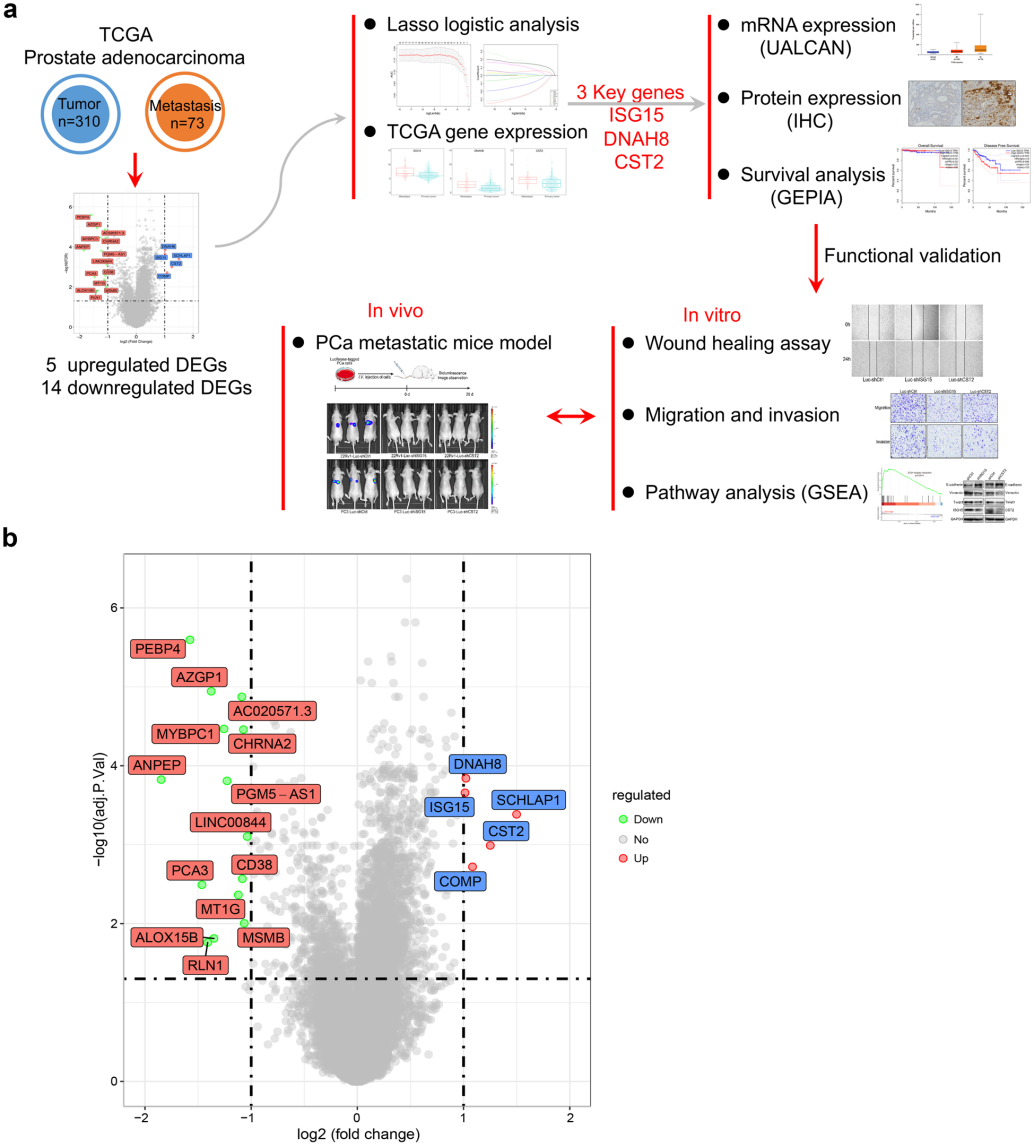
Identification of DEGs to compare primary and metastatic PCa from TCGA. **a** The flowchart of the present study aimed at identifying the novel key genes associated with the metastasis of PCa. **b** Volcano plot of DEGs identified based on the TCGA database. Red dots indicate upregulated genes, green dots indicate downregulation, and gray dots indicate no statistically significant difference

The procedure of the present study is shown in the following flowchart (Fig. 1a). First, a comparison between primary prostate adenocarcinoma tissues (n = 310) and metastatic prostate adenocarcinoma tissues (n = 71) was conducted to identify the DEGs responsible for PCa metastasis. The corresponding clinical information of the prostate adenocarcinoma samples is listed in Additional file 1: Table S4. A total of 19 DEGs were identified as specific molecules for PCa metastasis (Fig. 1b). Among them, five DEGs were upregulated, and 14 DEGs were downregulated (Fig. 1b).

**Fig. 2:**
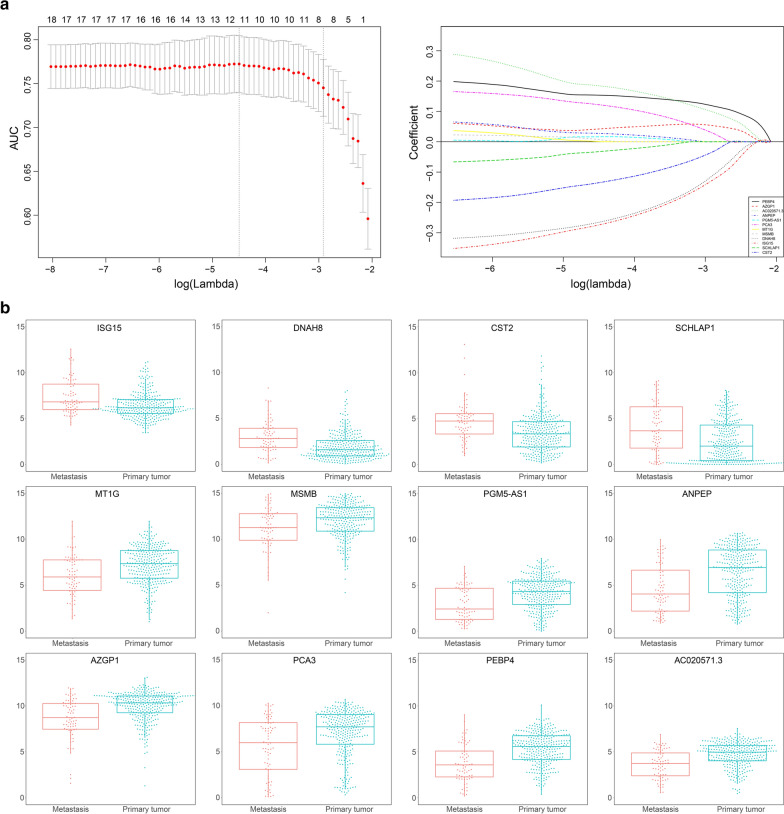
LASSO logistical analysis of DEGs. **a** LASSO logistical regression method was conducted to screen the pivotal PCa metastasis-related DEGs. **b** Expression of the pivotal PCa metastasis-related DEGs in TCGA dataset

To further investigate the importance of these DEGs, LASSO logistic regression was performed to select the key genes for PCa metastasis. Based on the analysis, 12 genes were found to be closely related to PCa prognosis (Fig. 2a) and their relative expression from TCGA database was shown in Fig. 2b. Among them, ubiquitin-like protein (ISG15), dynein axonemal heavy chain 8 (DNAH8), and cystatin SA (CST2) displayed the highest coefficient for PCa metastasis and were identified as the potential key genes (Additional file 1: Table S5).

### Expression of the potential key genes in metastatic PCa

**Fig. 3:**
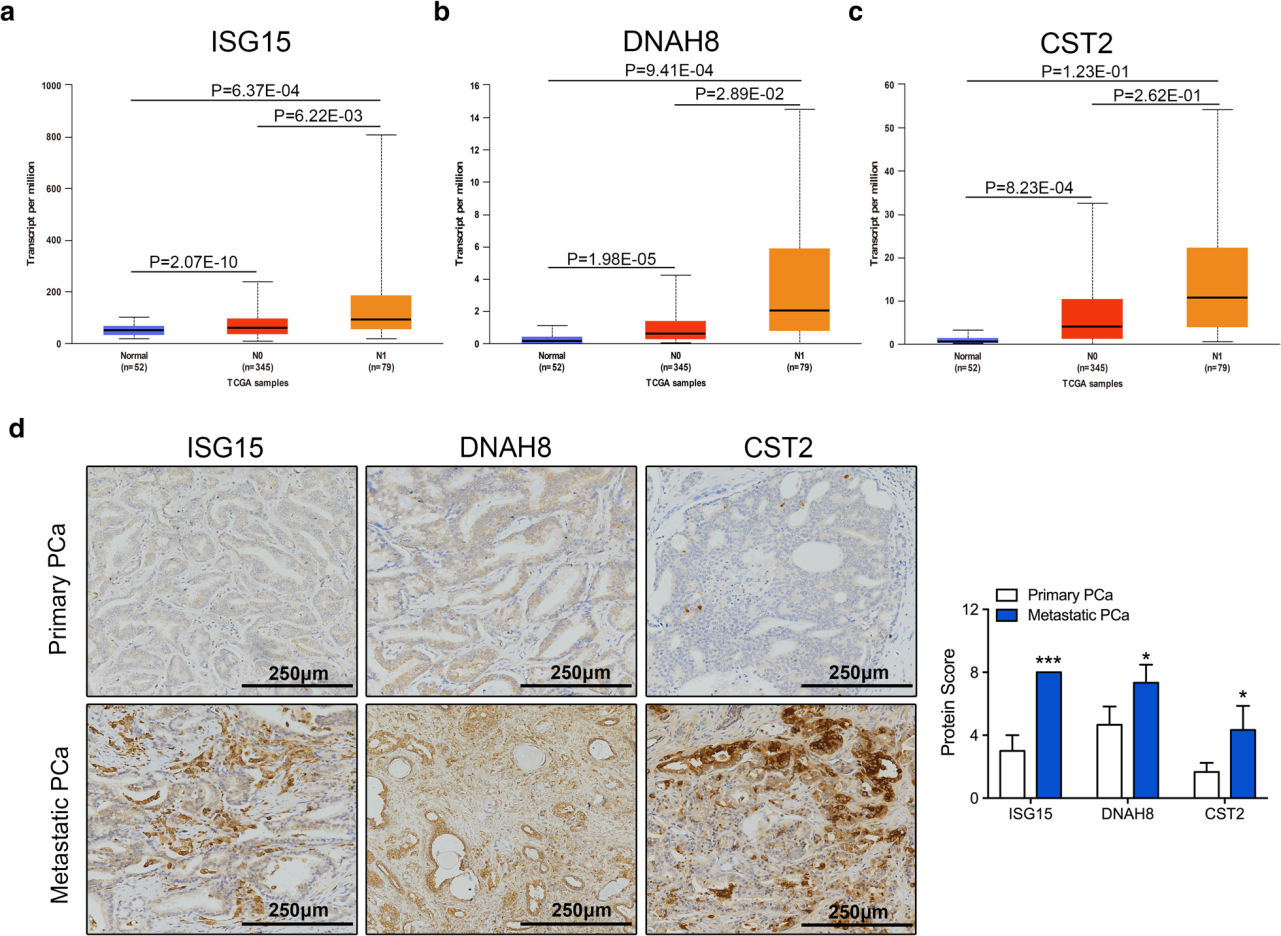
Expression of the three key genes in metastatic PCa. **a–c** Transcriptional expression of ISG15, DNAH8, and CST2 in metastatic, primary, and normal prostate tissues was performed using the UALCAN database. N0: No regional lymph node metastasis; N1: Metastases in 1 to 3 axillary lymph nodes. P < 0.05 was considered statistically significant. **d** Representative IHC images of ISG15, DNAH8, and CST2 in primary and metastatic prostate tissues (Scale: 250 mm)

After the pivotal genes were picked out in metastatic PCa, the transcriptional expression levels of these genes were obtained from the UALCAN database and the translational levels were determined by IHC. As shown in Fig. 3a–3c, mRNA expression of ISG15, DNAH8, and CST2 was significantly upregulated in metastatic prostate tissue samples. Moreover, the protein expression of these genes was also remarkably higher compared to that in primary tumor tissues (Fig. 3d).

### High expression of the potential key genes was associated with poor prognosis of PCa

**Fig. 4:**
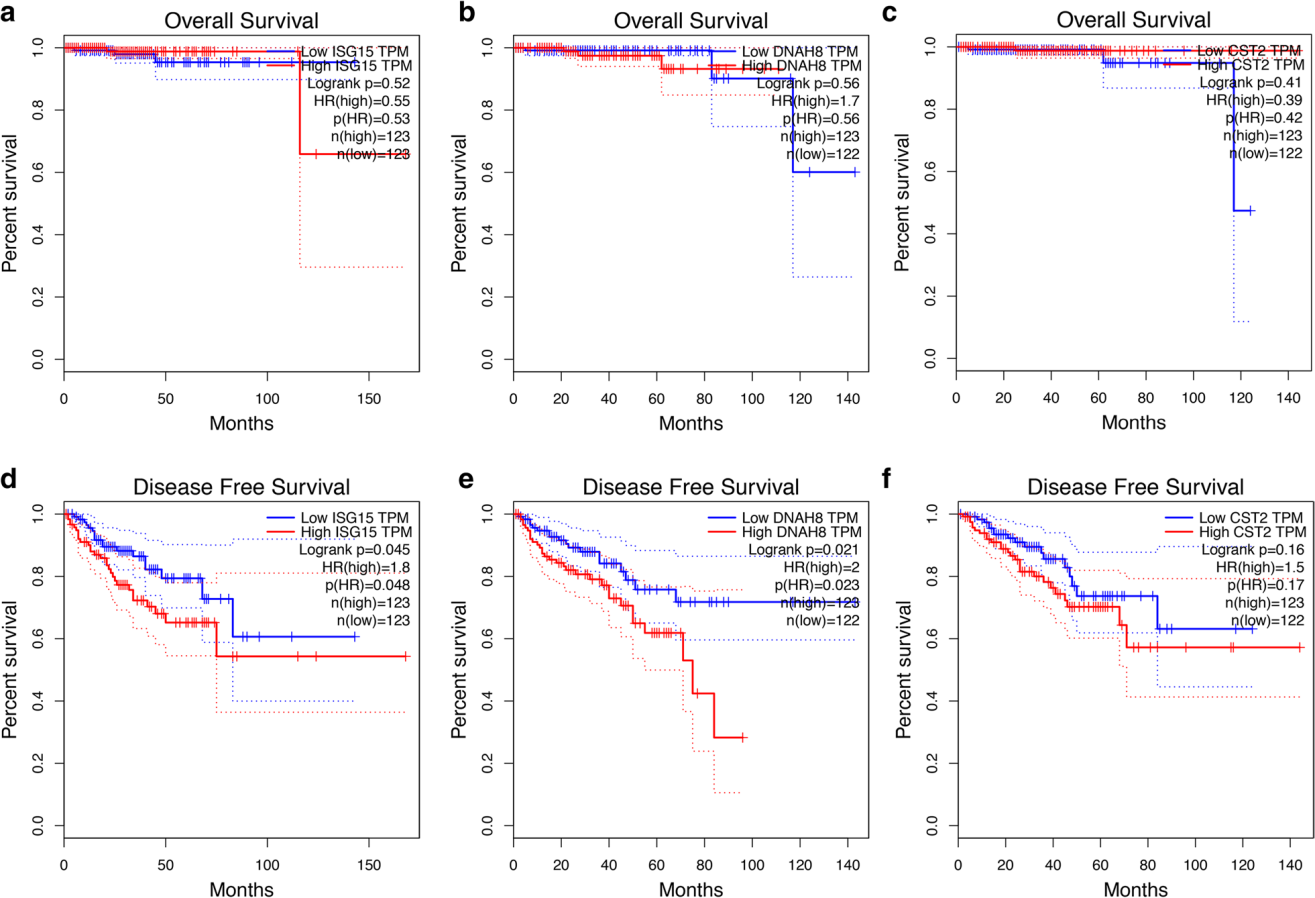
Prognostic analysis of the three key genes in metastatic PCa. **a–c** Overall survival analysis of ISG15, DNAH8, and CST2 in PCa patients was performed using the GEPIA database. **e–f** Disease-free survival analysis of ISG15, DNAH8, and CST2 in PCa patients was performed using the GEPIA database. P < 0.05 was considered statistically significant

To further clarify the association between these key genes and the progression of PCa, survival analysis to determine overall survival (OS) and disease-free survival (DFS) was performed using the GEPIA online database. With respect to ISG15 and DNAH8, expression changes of both genes had no effect on the OS of PCa patients, but significantly influenced the DFS of PCa patients (Fig. 4a, b, d, e). However, regarding CST2, there was no statistically significant difference in OS and DFS in PCa patients with CST2 high or low expression (Fig. 4c, f).

### Knockdown of the potential key genes inhibited the migration and invasion of PCa cells

**Fig. 5:**
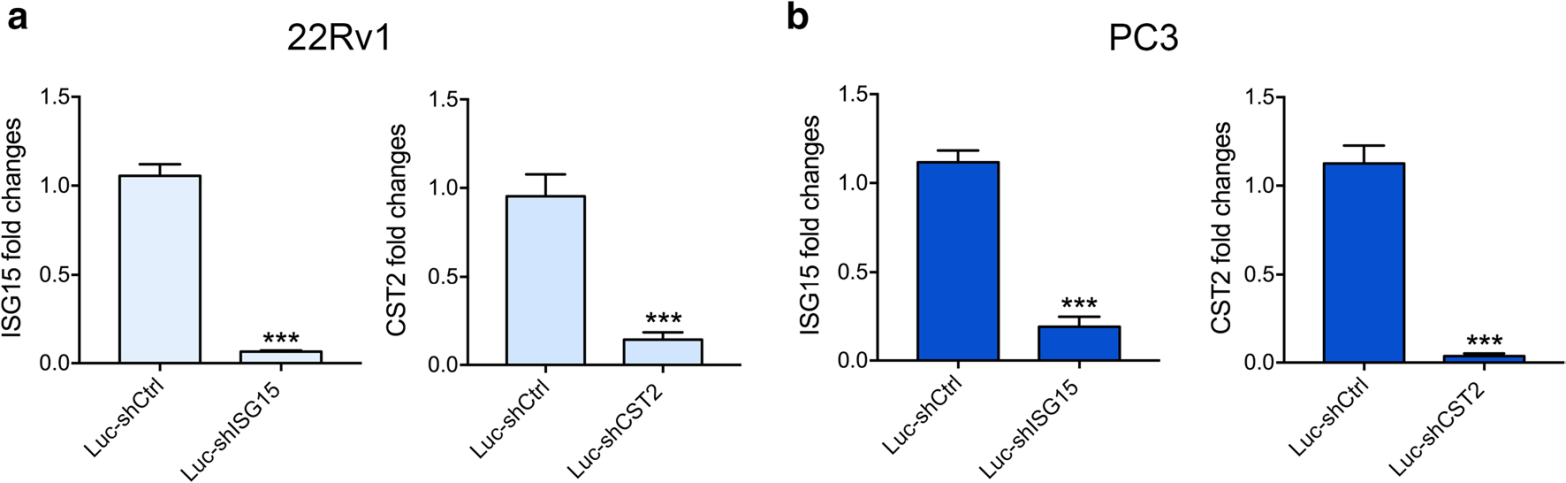
Knockdown of ISG15 and CST2 in PCa cells. **a, b** mRNA expression of ISG15 or CST2 in 22Rv1 and PC3 cells transfected with the corresponding shRNA for 48 h and determined by qRT-PCR. Data are shown as mean ± SD, ^*^P < 0.05, ^**^P < 0.01, ^***^P < 0.001

**Fig. 6:**
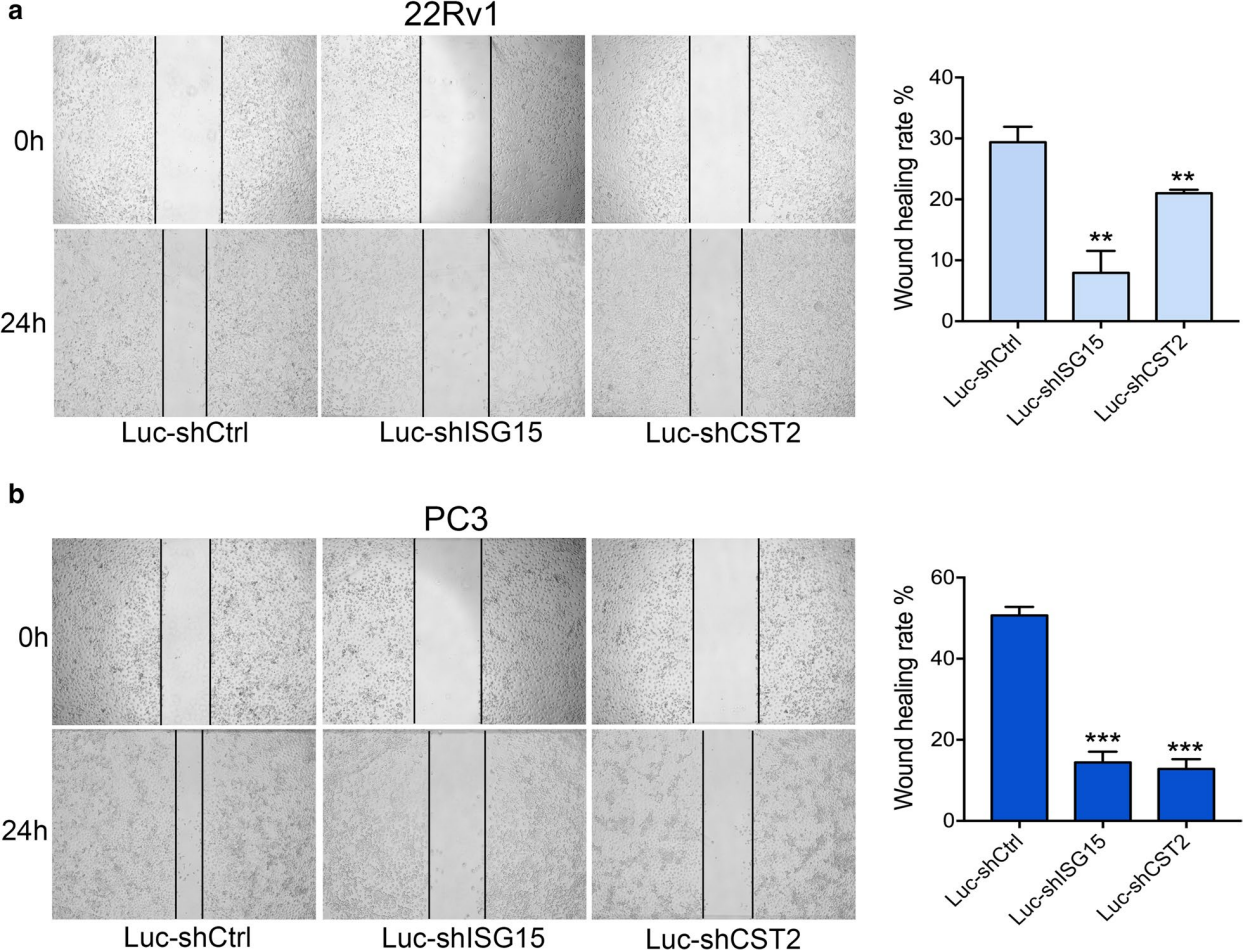
Wound healing assay of ISG15 and CST2 knockdown in PCa cells. **a, b** Wound healing assay of ISG15 and CST2 knockdown was conducted in 22Rv1 and PC3 cells. Data are shown as mean ± SD, *P < 0.05, **P < 0.01, ***P < 0.001

**Fig. 7:**
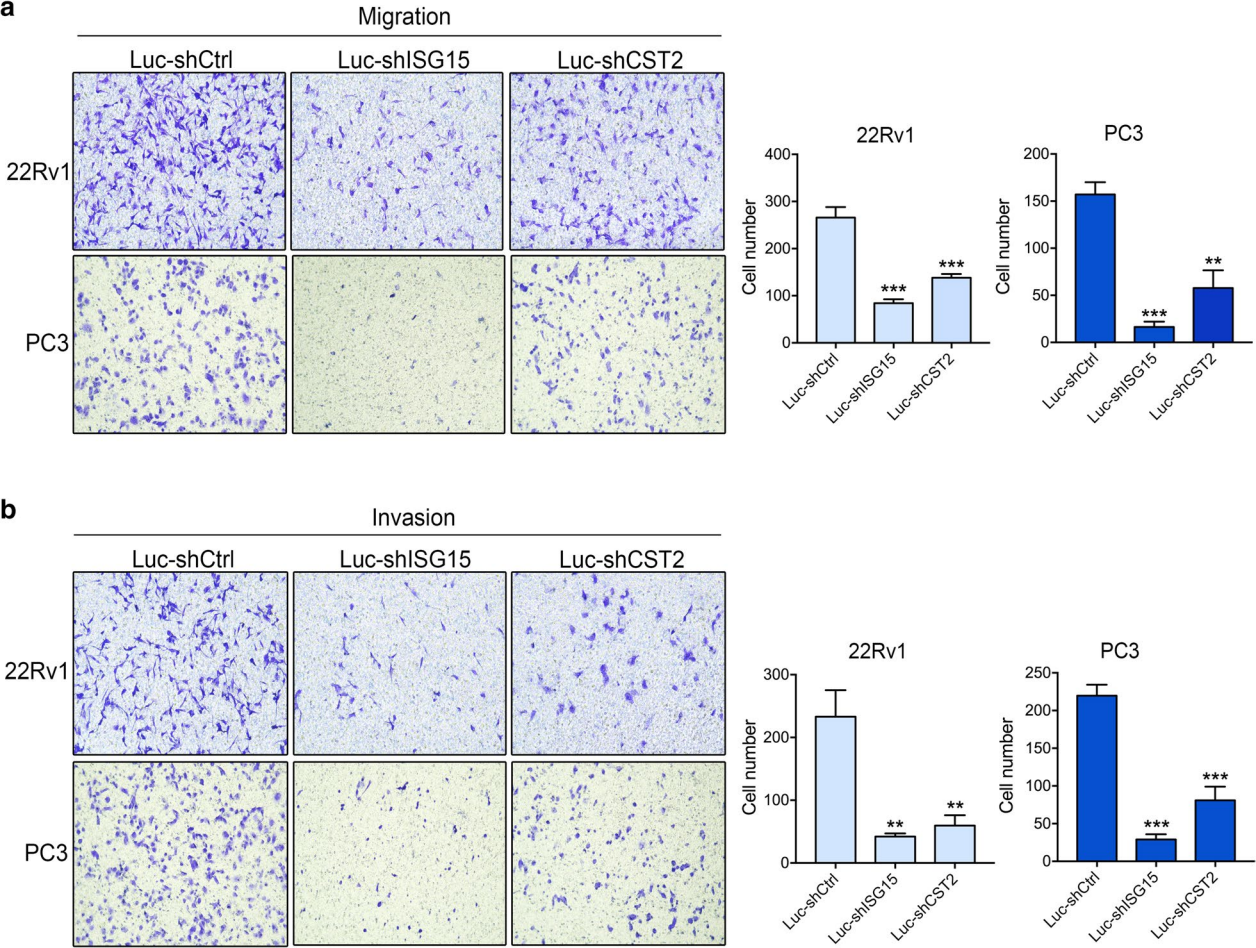
ISG15 and CST2 influence the migration and invasion of PCa cells. **a** Migration of 22Rv1 and PC3 cells with or without ISG15 and CST2 knockdown. **b** Invasion of 22Rv1 and PC3 cells with or without ISG15 and CST2 knockdown. Data are shown as mean ± SD, *P < 0.05, **P < 0.01, ***P < 0.001

To further confirm the role of the potential key genes in metastasis, we evaluated the influence of migration and invasion by gene knockdown in PCa cells. First, the knockdown efficiency of the three genes was evaluated at the mRNA levels. As shown in Fig. 5, shRNA of ISG15 and CST2 was successfully transfected into 22Rv1 and PC3 cells, with a knockdown efficiency of greater than 85%. However, the expression of DNAH8 was undetectable in five different prostate cancer cell lines. Therefore, a Transwell assay was conducted to determine the inhibition of migration and invasion by knockdown of ISG15 and CST2. Our results showed that knockdown of ISG15 and CST2 markedly suppressed wound healing in both cell lines (Fig. 6). Moreover, ISG15 and CST2 knockdown greatly decreased the number of PCa cells passing through the membrane (Fig. 7).

### Potential key genes played important roles in regulating metastasis-associated pathways

**Fig. 8:**
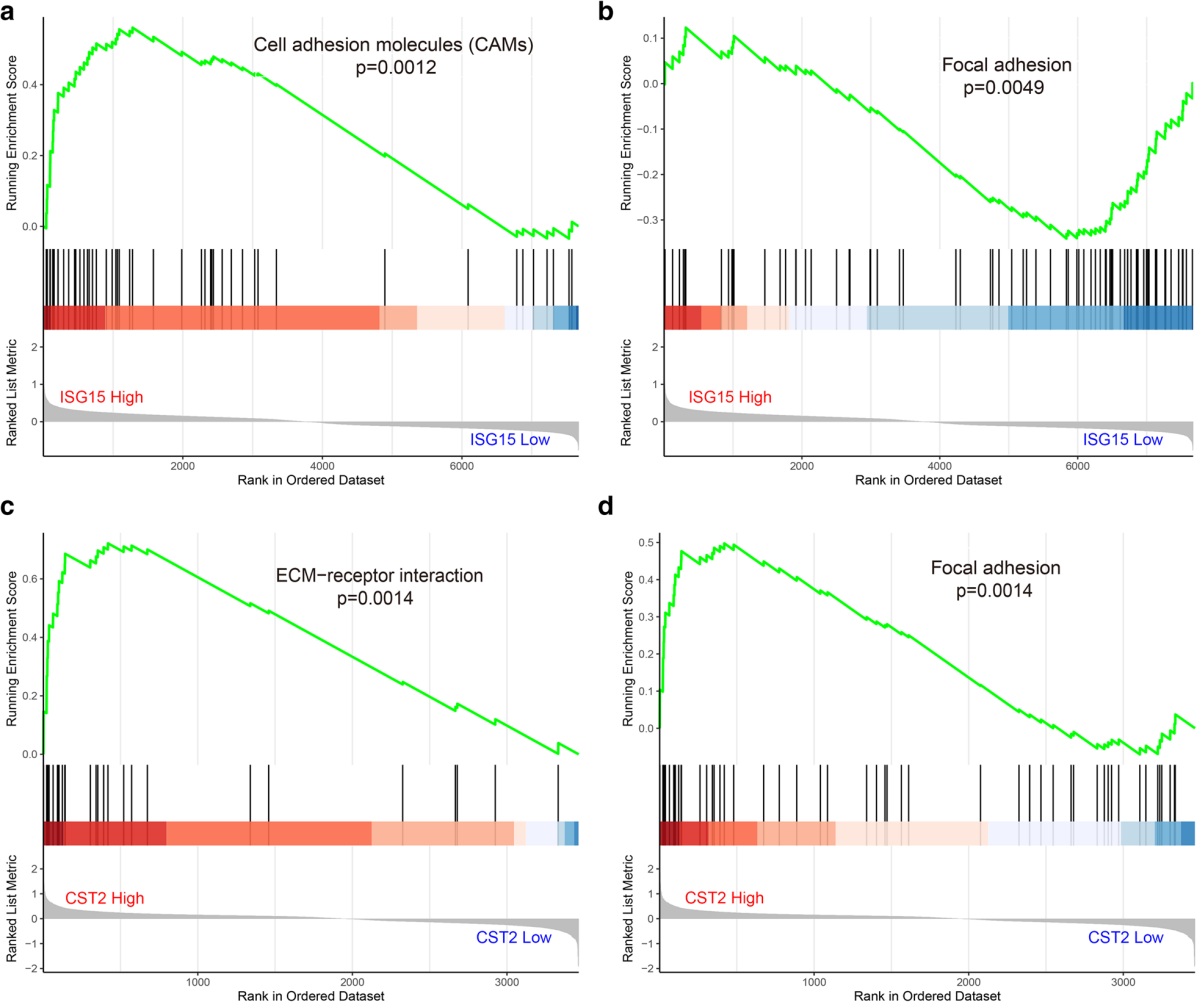
ISG15 and CST2-mediated signaling pathway for PCa progression. **a, b** GSEA was used to perform the KEGG pathway enrichment grouped by ISG15 expression level in TCGA dataset. **c, d** GSEA was used to perform the KEGG pathway enrichment grouped by CST2 expression level in TCGA dataset

To further characterize the pathway of these genes, GSEA was conducted to perform the pathway enrichment analysis. Our data showed that ISG15 was closely associated with cell adhesion molecules (CAMs) and focal adhesion (Fig. 8a, b). Changes in the expression of CST2 were mainly enriched in extracellular matrix (ECM)-receptor interaction and focal adhesion (Fig. 8c, d). These data indicated that ISG15 and CST2 might regulate epithelial matrix transformation to influence PCa metastasis.

### Potential key genes influenced the metastasis of PCa by regulating the epithelial-mesenchymal transition (EMT) pathway

**Fig. 9:**
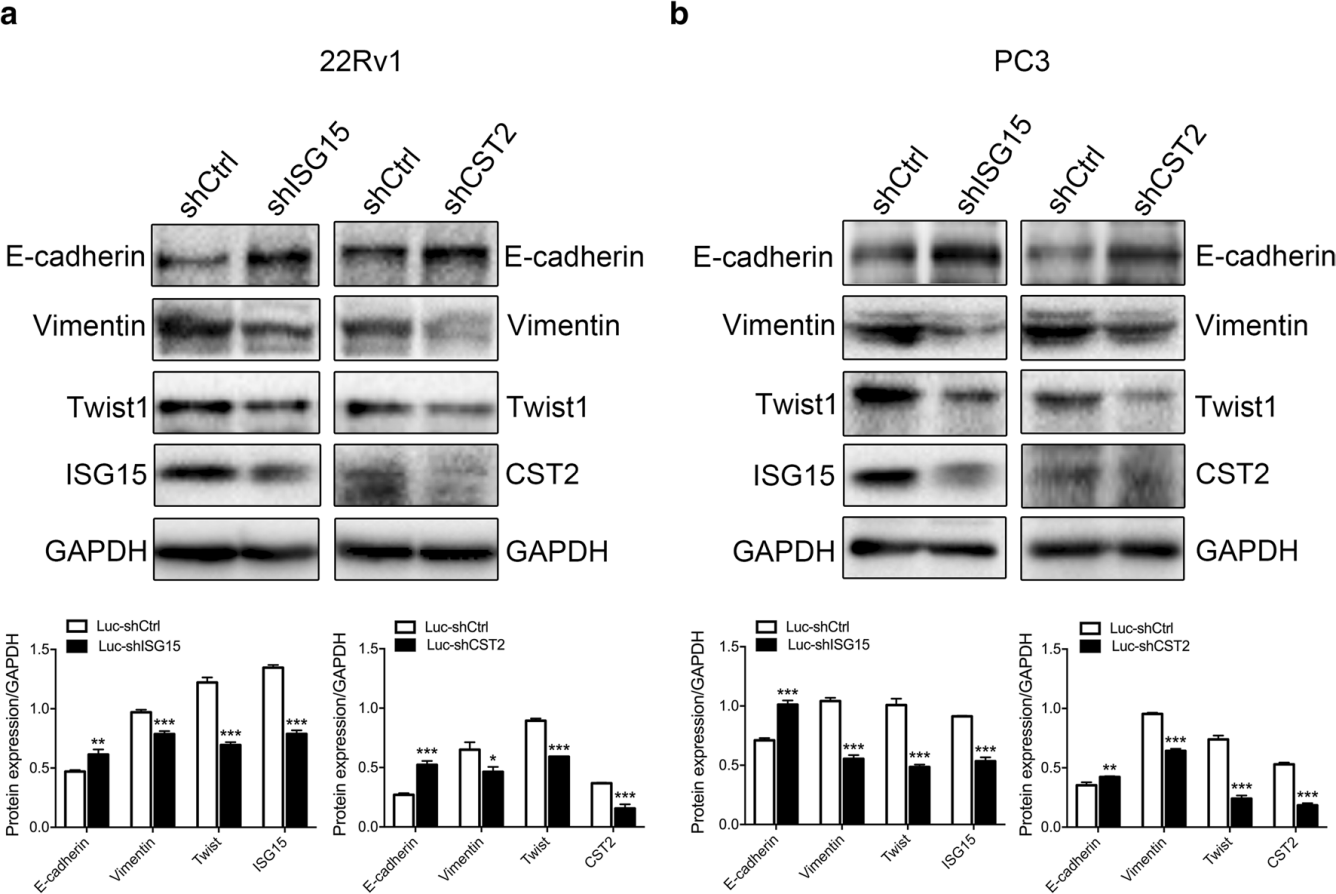
ISG15 and CST2 influence PCa metastasis through regulating EMT signaling pathway. **a** Protein expression of E-cadherin, Twist1, and Vimentin in 22Rv1 cells with or without ISG15 and CST2 knockdown. **b** Protein expression of E-cadherin, Twist1, and Vimentin in PC3 cells with or without ISG15 and CST2 knockdown. Data were shown as mean ± SD (n = 3), *P < 0.05, **P < 0.01, ***P < 0.001

Since ISG15 and CST2 knockdown influenced the migration and invasion of PCa cells, we determined the expression of EMT-associated proteins with ISG15 or CST2 knockdown. Our results demonstrated that the expression of E-cadherin significantly increased when ISG15 or CST2 was knocked down (Fig. 9). Moreover, knockdown of ISG15 or CST2 decreased the protein expression of Twist1 and Vimentin. These findings indicated that ISG15 and CST2 could regulate the EMT signaling pathway, thereby facilitating PCa metastasis.

### Knockdown of the key genes repress the metastasis of PCa cells in vivo

**Fig. 10:**
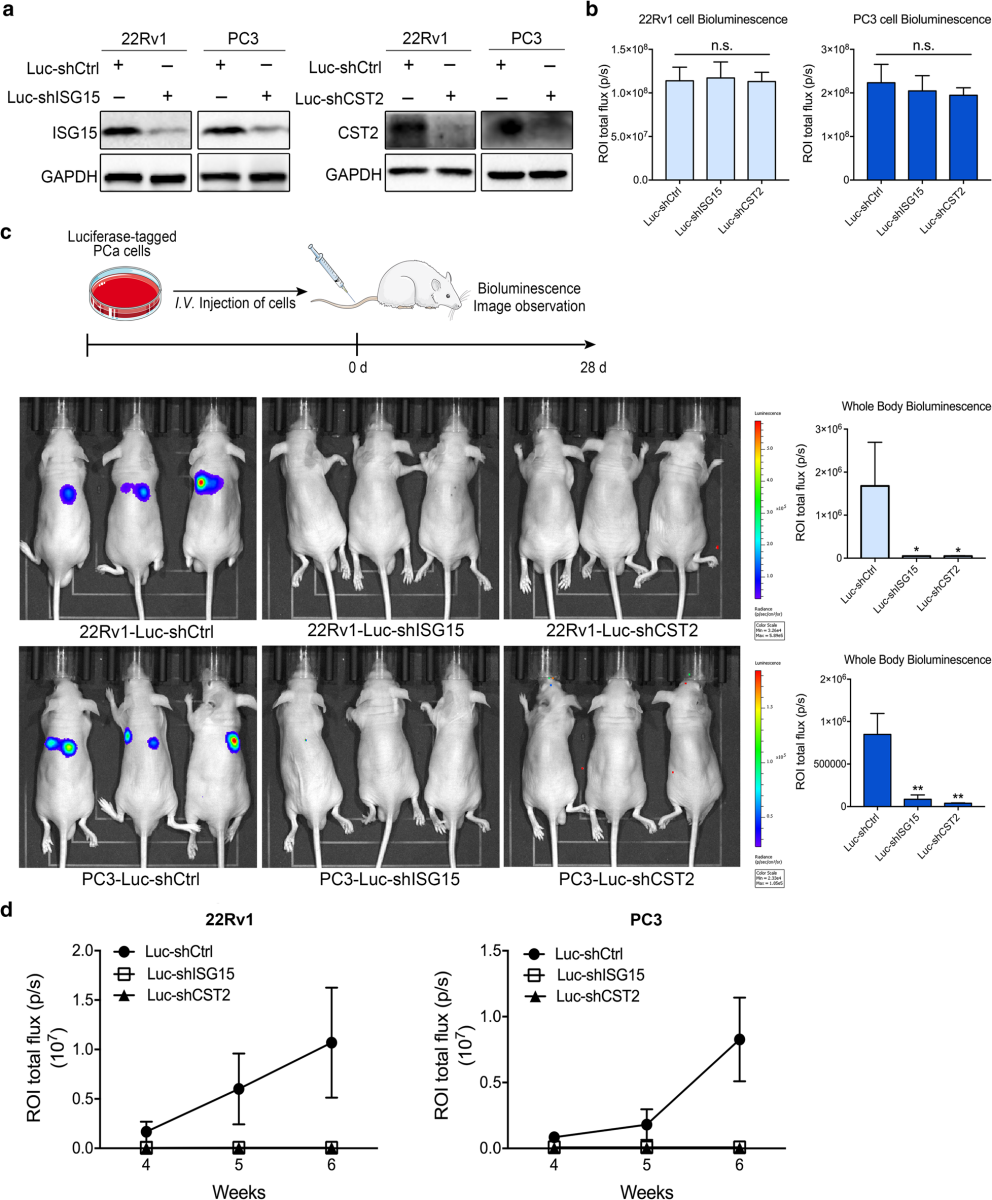
ISG15 and CST2 influence PCa cells metastasis in vivo. **a** Protein expression of ISG15 and CST2 in PC3 and 22Rv1 stable cell lines. **b** The intensities of region of interest (ROI) were assessed in PC3 and 22Rv1 stable cell lines, *n.s.* no difference. **c** Bioluminescence images of tumor cell metastasis at 4 weeks. Color scale bar represents the intensity of the tumor from violet (low) to red (high). right panel: quantification of whole animal bioluminescence and plotted as total flux (photons/sec). **d** Bioluminescence images curves of the development of tumor cells metastases in shCtrl, shISG15 and shCST2 group. Data are shown as mean ± SD, *P < 0.05, **P < 0.01, ***P < 0.001

To further characterized the role of ISG15 and CST2 in PCa metastasis in vivo, we established PCa cells stably expressing luciferase-22Rv1-shCtrl, 22Rv1-shISG15, 22Rv1-shCST2, PC3-shCtrl, PC3-sh ISG15, or PC3-sh CST2. The protein expression and luciferase activities were firstly measured to confirm the key genes were stably expressed in both PCa cells (Fig. 10a, b). Tumor cells in different groups were injected into mice through the intravenous route, and the distribution of tumor cells was monitored by bioluminescence images (Fig. 10c). Compared with mice in the shCtrl group, mice in the shISG15 or shCST2 group failed to exhibit any signs of tissue metastases in both 22Rv1 and PC3 cells, even when the observation period was extended to 6 weeks (Fig. 10c, d). These findings strongly indicated that ISG15 and CST2 were crucial for PCa metastasis.

## Discussion

Metastatic PCa is a lethal malignant tumor with a low 5-year survival rate, which seriously influences patients’ quality of life. However, effective therapeutic targets for PCa metastasis remain scarce; thus, identifying novel key genes implicated in PCa metastasis and elucidating their underlying mechanisms are required.

In the present study, we analyzed the TCGA database and screened 19 DEGs (five upregulated DEGs and 14 downregulated DEGs) between primary and metastatic PCa. Among them, ISG15, DNAH8, and CST2 were identified as key genes for PCa metastasis based on LASSO logistical analysis, and the expression of these genes in metastatic PCa samples was significantly higher than that in primary tumors. Moreover, PCa patients with high expression of these three key genes displayed worse OS or DFS. To further verify the effects of the three key genes on PCa metastasis, we knocked down the levels of ISG15 and CST2 in 22Rv1 and PC3 cells and conducted wound healing and Transwell assays and found that ISG15 and CST2 could dramatically suppress the migration and invasion of PCa cells. Additionally, GSEA showed that CAMs, ECM-receptor interaction, and focal adhesion were mainly enriched in the ISG15 or CST2 high expression group. Thus, we investigated the expression of EMT-related proteins in PCa cells with ISG15 or CST2 knockdown. Western blotting results demonstrated that the expression levels of E-cadherin increased in the ISG15 and CST2 knockdown groups, whereas the expression of Twist1 and Vimentin significantly decreased when ISG15 and CST2 were knocked down. Furthermore, knockdown of ISG15 and CST2 could significantly inhibit the PCa cells metastasis in vivo. These findings indicate that ISG15 and CST2 can promote PCa metastasis by regulating EMT, which may be potential targets for metastatic PCa therapy.

Interferon-stimulated gene 15 (SG15) is a small ubiquitin-like protein that is induced by type I interferons and plays an important role in regulating the innate immune responses to viral or bacterial infection [20, 21]. Currently, numerous studies have reported that ISG15 is involved in various tumors, such as breast cancer, oral squamous cell carcinoma, pancreatic cancer, and endometrioid endometrial adenocarcinoma [22–25]. Satake et al. have found that ISG15 expression in high-grade PCa was significantly higher than that in low-grade PCa and normal prostate tissues [26]. Chen et al. have revealed that ISG15 was highly expressed in LN1-1 cells, and its increased expression could promote the lymphatic metastasis of oral squamous cell carcinoma by interacting with Rac1 [22]. Lo et al. have demonstrated that knockdown of ISG15 substantially inhibited the migration and invasion of breast cancer cells and reduced the expression of EMT programming genes, suggesting that ISG15 is important for maintaining the malignancies of triple-negative breast cancer [27]. Meanwhile, ISG15 has been identified as a novel diagnostic biomarker of thyroid papillary microcarcinoma with lymphatic metastasis through proteomic analysis [28]. Further analysis has found that ISG15 could be secreted by nasopharyngeal carcinoma cells and induced macrophages to M2 polarization, thereby inhibiting the antitumor activity of T cells and accelerating tumor progression, indicating that ISG15 is an important molecule in tumor microenvironment [29]. However, ISG15 plays a role not only in promoting tumor progression but also in preventing tumor development. A recent study has reported that high expression of ISG15 was correlated with a better prognosis in patients with lung adenocarcinoma [30]. Although numerous studies have demonstrated that ISG15 is essential for tumor metastasis, its function in PCa metastasis and the underlying molecular mechanism are not fully understood. Here, we found that ISG15 was upregulated in metastatic PCa and its knockdown inhibited the migration and invasion of prostate cells in vitro and in vivo. Moreover, inhibition of ISG15 led to increase the expression of E-cadherin and decrease the expression of Twist1 and Vimentin. These findings indicate that ISG15 may be a promising target for the therapy of PCa progression.

DNAH8 is a heavy chain of an axoneme, and its role in PCa or other tumors is largely unknown. To date, the function of DNAH8 is mainly involved in sperm flagellum and respiratory cilia motility. Liu et al. have illustrated that double allelic mutation of DNAH8 induced multiple morphological abnormalities of the flagella, leading to infertility in men [31]. Exome sequencing analysis has revealed that DNAH8 variants were significantly increased in patients with chronic obstructive pulmonary disease when compared to resistant smokers [32]. Nevertheless, only one study by Wang et al. has reported that DNAH8 was upregulated in metastatic PCa tissues, and patients with high expression of DNAH8 were more likely to experience tumor relapse and metastasis than their counterparts [33]. In our study, we also tried to knock down DNAH8 in PC3, 22Rv1, DU145, LNCaP, and C4-2 cell lines; however, the transcriptional expression of DNAH8 in all the cell lines was not detectable. Based on these results, we hypothesized that the primers were not suitable for DNAH8. Therefore, we designed ten qRT-PCR primers and also included a primer reported by Wang et al. to determine the mRNA expression of DNAH8 in prostate cells [33]. Unfortunately, DNAH8 was still undetectable using all primers. Since then, we have ended the investigation of DNAH8 in PCa metastasis.

CST2 is a cysteine proteinase inhibitor that belongs to the cystatin superfamily. Studies regarding its function in cancer are limited, especially in PCa or metastatic PCa. A previous study has detected the CST2 expression and found that CST2 was aberrantly higher in HCC tissues than in normal controls [34]. Moreover, a global secretome analysis has demonstrated that elevated expression of CST2 could promote bone metastasis in breast and bladder cancers [35]. Additionally, a recent study revealed that CST2 was upregulated in gastric cancer and was positively correlated with the poor prognosis of patients with gastric cancer, and increased CST2 expression could enhance the proliferation, migration, and invasion of gastric cells through regulating the EMT signaling pathway [36]. Interestingly, Cheng et al. have found that patients with high CST2 expression were more likely to develop aggressive PCa than their counterparts. These studies indicate that CST2 may act as an important protein in tumor metastasis. However, the association between CST2 and metastatic PCa and its underlying mechanism has not been fully elucidated. In the present study, we found that the prognosis of PCa patients with CST2 high or low expression showed no significant difference. However, knockdown of CST2 in PCa cells could inhibit cell migration and invasion in vitro and in vivo, whose function was to abolish the EMT pathway. Therefore, whether CST2 is an important factor for the development of PCa requires more clinical samples and experimental data.

In the present study, we identified three key genes involved in metastatic PCa through integrated bioinformatics analysis and experimental validation. These key genes may not only be used to elucidate the pathogenic mechanism of advanced PCa but also be used as potential therapeutic targets for this cancer. However, the specific function of these key genes in PCa progression has not been fully understood, as the results obtained in this study were based on bioinformatics analysis of public databases, and experiments were only performed with wound healing and Transwell assays, ETM-related protein expression and in vivo metastatic mice model. Therefore, we will collect a large number of clinical metastatic PCa tissue samples, utilize western blotting and immunohistochemistry to further characterize the molecular mechanisms and screen the candidate drugs to verify the possibility of these key genes as therapeutic targets in the future.

## Conclusions

In summary, we screened 19 DEGs (five upregulated DEGs and 14 downregulated DEGs) to compare primary and metastatic PCa. According to the LASSO logistical analysis, the following genes were identified that might be pivotal PCa metastasis-related key genes: ISG15, DNAH8, and CST2. Further functional experiments revealed that knockdown of ISG15 and CST2 could suppress the metastasis and invasion of PCa cells and influence the protein expression of genes in the EMT signaling pathway. These findings provide new insights for better understanding the progression and molecular mechanism of PCa and might offer candidate therapeutic targets for PCa metastasis.

## Supplementary Information

**Additional file 1: Table S1**. Clinical information on prostate cancer patients. **Table S2**. shRNA sequences used in this study. **Table S3**. Sequences of qRT-PCR primers used in this study. **Table S4**. Clinicopathological characteristics of PCa patients from TCGA database. **Table S5**. Coefficients of DEGs identified in metastatic PCa based on LASSO logistic analysis.

## Data Availability

The datasets used and analyzed during the present study are available from the corresponding author on reasonable request.

## Abbreviations

ADT: Androgen deprivation therapy
CAMs: Cell adhesion molecules
CST2: Cystatin 2
CXCL1: Chemokine (C-X-C motif) ligand 1
DEG: Differentially expressed gene
DFS: Disease-free survival
DNAH8: Dynein axonemal heavy chain 8
ECMs: Extracellular matrices
EMT: Epithelial-mesenchymal transition
EZH2: Enhancer of zeste homolog 2
FPKM: Fragments per kilobase
GEPIA: Gene Expression Profiling Interactive Analysis
GSEA: Gene set enrichment analysis
IHC: Immunohistochemistry
ISG15: Interferon-stimulated gene 15
KEGG: Kyoto Encyclopedia of Genes and Genomes
LASSO: Least absolute shrinkage and selection operator
OS: Overall survival
PBS: Phosphate buffered saline
PMSF: Phenylmethylsulfonyl fluoride
PVDF: Polyvinylidene fluoride
qRT-PCR: Real-time quantitative PCR
RIPA: Radio-immunoprecipitation Assay
SD: Standard deviation
SDS-PAGE: Sodium dodecyl sulfate polyacrylamide gel electrophoresis
TBST: Tris-buffered saline with Tween 20
TCGA: The Cancer Genome Atlas
TPM: Transcripts per million
