# Supplementary material for: Identification of novel key genes associated with the metastasis of prostate cancer based on bioinformatics prediction and validation
Source: Cancer Cell Int. 2021 Oct 25;21:559. doi: 10.1186/s12935-021-02258-3 (PMC8547030; doi:10.1186/s12935-021-02258-3)
Supplement: Supplementary file 1 — Additional file 1: Table S1. Clinical information on prostate cancer patients. Table S2. shRNA sequences used in this study. Table S3. Sequences of qRT-PCR primers used in this study. Table S4. Clinicopathological characteristics of PCa patients from TCGA database. Table S5. Coefficients of DEGs identified in metastatic PCa based on LASSO logistic analysis. [file 12935_2021_2258_MOESM1_ESM.docx]

Table S1. Clinical information on prostate cancer patients.

| No. | Age | Sex | Organ | Tissue type | Gleason score | TNM |
| --- | --- | --- | --- | --- | --- | --- |
| 1 | 71 | Male | Prostate | adenocarcinoma | 7 | T2cN0M0 |
| 2 | 74 | Male | Prostate | adenocarcinoma | 7 | T2aN0M0 |
| 3 | 67 | Male | Prostate | adenocarcinoma | 7 | T2bN0M0 |
| 4 | 56 | Male | Prostate | adenocarcinoma | 9 | T3bN1Mx |
| 5 | 63 | Male | Prostate | adenocarcinoma | 9 | T3aN1Mx |
| 6 | 75 | Male | Prostate | adenocarcinoma | 8 | T3bN1Mx |

Table S2. shRNA sequences used in this study.

| Gene | Sequence |
| --- | --- |
| ISG15 | CCGGCTGAGCATCCTGGTGAGGAATCTCGAGATTCCTCACCAGGATGCT  CAGTTTTT |
| CST2 | CCGGGTGAATTACTTCTTCGACATACTCGAGTATGTCGAAGAAGTAATTC  ACTTTTT |

Table S3. Sequences of qRT-PCR primers used in this study.

| Gene | Primer sequence |
| --- | --- |
| human ISG15 | Forward: GTGGACAAATGCGACGAACC |
|  | Reverse: ATTTCCGGCCCTTGATCCTG |
| human CST2 | Forward: AGCGTGCCCTTCACTTTGTCAT |
|  | Reverse: TACATATGGTTCGGCCCACCTCT |
| human GAPDH | Forward: GCACCGTCAAGGCTGAGAAC |
|  | Reverse: TGGTGAAGACGCCAGTGGA |

Table S4. Clinicopathological characteristics of PCa patients from TCGA database

|  | Level | Metastasis | Primary Tumor |
| --- | --- | --- | --- |
| n |  | 73 | 310 |
| Clinical_M (%) | M0 | 71 (97.3) | 310 (100.0) |
|  | M1a | 1 (1.4) | 0 (0.0) |
|  | M1c | 1 (1.4) | 0 (0.0) |
| Pathologic_N (%) | N0 | 1 (1.4) | 310 (100.0) |
|  | N1 | 72 (98.6) | 0 (0.0) |
| Pathologic_T (%) | T2a | 0 (0.0) | 7 (2.3) |
|  | T2b | 0 (0.0) | 8 (2.6) |
|  | T2c | 2 (2.7) | 115 (37.1) |
|  | T3a | 16 (21.9) | 115 (37.1) |
|  | T3b | 51 (69.9) | 58 (18.7) |
|  | T4 | 4 (5.5) | 4 (1.3) |
|  | unknown | 0 (0.0) | 3 (1.0) |
| Age (%) | <60 | 32 (43.8) | 119 (38.4) |
|  | >=60 | 41 (56.2) | 191 (61.6) |
| PSA (%) | <=20 | 3 (4.1) | 7 (2.3) |
|  | <10 | 59 (80.8) | 280 (90.3) |
|  | >20 | 11 (15.1) | 23 (7.4) |
| Gleason_score (%) | 6 | 0 (0.0) | 19 (6.1) |
|  | 7 | 12 (16.4) | 179 (57.7) |
|  | 8 | 14 (19.2) | 40 (12.9) |
|  | 9 | 46 (63.0) | 71 (22.9) |
|  | 10 | 1 (1.4) | 1 (0.3) |
| Gleason_score.1 (%) | <7 | 0 (0.0) | 19 (6.1) |
|  | >=7 | 61 (83.6) | 112 (36.1) |
|  | 7 | 12 (16.4) | 179 (57.7) |
| Laterality (%) | Bilateral | 68 (93.2) | 271 (87.4) |
|  | Left | 2 (2.7) | 12 (3.9) |
|  | Right | 3 (4.1) | 20 (6.5) |
|  | unknown | 0 (0.0) | 7 (2.3) |

Table S5. Coefficients of DEGs identified in metastatic PCa based on LASSO logistic analysis

|  | Gene | Coefficients |
| --- | --- | --- |
|  | ISG15 | -0.27408221012823 |
|  | DNAH8 | -0.265278276115616 |
|  | CST2 | -0.134964775889836 |
|  | SCHLAP1 | -0.0325459423848736 |
|  | MT1G | 0.000505833013903387 |
|  | MSMB | 0.00710275725436536 |
|  | PGM5.AS1 | 0.0164910414172834 |
|  | ANPEP | 0.0274456424871278 |
|  | AZGP1 | 0.0421112175142419 |
|  | PCA3 | 0.12196832557356 |
|  | PEBP4 | 0.15337413605244 |
|  | AC020571.1 | 0.184210423149045 |
